# Proteomic Analysis, Immuno-Specificity and Neutralization Efficacy of Pakistani Viper Antivenom (PVAV), a Bivalent Anti-Viperid Antivenom Produced in Pakistan

**DOI:** 10.3390/toxins15040265

**Published:** 2023-04-03

**Authors:** Andy Shing Seng Lim, Kae Yi Tan, Naeem H. Quraishi, Saud Farooque, Zahoor Ahmed Khoso, Kavi Ratanabanangkoon, Choo Hock Tan

**Affiliations:** 1 Venom Research and Toxicology Laboratory, Department of Pharmacology, Faculty of Medicine, University of Malaya, Kuala Lumpur 50603, Malaysia; andy_lim0304@yahoo.com; 2 Protein and Interactomics Laboratory, Department of Molecular Medicine, Faculty of Medicine, University of Malaya, Kuala Lumpur 50603, Malaysia; kytan_kae@um.edu.my; 3 Snake Antivenom/Antirabies Serology Laboratory, Department of Community Medicine & Public Health Sciences, People’s University of Medical and Health Sciences for Women, Nawabshah 67450, Pakistan; naimquraishi@yahoo.com (N.H.Q.); saud.kk02@gmail.com (S.F.);; 4 Department of Microbiology, Faculty of Science, Mahidol University, Bangkok 73170, Thailand

**Keywords:** snakebite envenoming, immunoglobulin, antibody

## Abstract

Snakebite envenoming is a neglected tropical disease prevalent in South Asia. In Pakistan, antivenoms are commonly imported from India despite the controversy over their effectiveness. To solve the problem, the locals have developed the Pakistani Viper Antivenom (PVAV), raised against Sochurek’s Saw-scaled Viper (*Echis carinatus sochureki*) and Russell’s Viper (*Daboia russelii*) of Pakistani origin. This study is set to evaluate the composition purity, immuno-specificity and neutralization efficacy of PVAV. Chromatographic and electrophoretic profiling coupled with proteomic mass spectrometry analysis showed PVAV containing high-purity immunoglobulin G with minimum impurities, notably the absence of serum albumin. PVAV is highly immuno-specific toward the venoms of the two vipers and *Echis carinatus multisquamatus*, which are indigenous to Pakistan. Its immunoreactivity, however, reduces toward the venoms of other *Echis carinatus* subspecies and *D. russelii* from South India as well as Sri Lanka. Meanwhile, its non-specific binding activities for the venoms of Hump-nosed Pit Vipers, Indian Cobras and kraits were extremely low. In the neutralization study, PVAV effectively mitigated the hemotoxic and lethal effects of the Pakistani viper venoms, tested *in vitro* and *in vivo*. Together, the findings suggest the potential utility of PVAV as a new domestic antivenom for the treatment of viperid envenoming in Pakistan.

## 1. Introduction

Snakebite envenoming is a global public health problem designated by the World Health Organization as a priority neglected tropical disease (NTD) [1]. Annually, snakebite envenoming causes 81,000–138,000 deaths and approximately 400,000 disabilities due to amputations and various chronic complications [2,3,4]. Rural populations in developing tropical countries are most heavily affected, particularly in South Asia [4,5]. India has the highest incidence and mortality of snakebites, where at least 80,000 cases and 11,000–46,000 deaths are reported yearly [3,4,6]. Snakebite envenoming is also prevalent in Pakistan, another South Asian country northwest of India, with an estimation of at least 40,000 bites that result in 1000–8200 deaths every year [3,7,8]. However, the number of snakebite cases in these regions is likely to be under-reported since a proper reporting system from remote areas is often non-existent. Snake species responsible for the mortality and morbidity of envenoming cases in Pakistan include Saw-scaled Vipers (*Echis carinatus* subspecies), Western Russell’s Viper (*Daboia russelii*), Sind Krait (*Bungarus sindanus*), Common Krait (*Bungarus caeruleus*), and Pakistani Black Cobra or Indian Cobra (*Naja naja*).

The definitive treatment for snakebite envenoming is the timely administration of safe and effective antivenom [9]. Antivenoms are usually produced at small scales for use within a country or a limited geographical region due to the high production cost and small market. Consequently, the antivenom supply is tight and inconsistent in many parts of the world. In South Asia, most countries rely on antivenoms produced in and imported from India since the major venomous snake species found throughout the region are somewhat similar, i.e., the Big Four that includes *N. naja*, *B. caeruleus*, *D. russelii* and *E. carinatus*. However, antivenoms imported from India were shown to be less effective against the venom toxicity of snakes in other countries, such as Pakistan and Sri Lanka [10,11,12,13,14,15,16]. This could be due to geographical venom variability in a snake species, where variation in venom composition is accompanied by differences in toxin antigenicity, and the resulting low efficacy of imported antivenoms [14,15,17]. Moreover, although the Indian antivenoms are raised against the Big Four species, these products are not indicated for other endemic snake species, such as the Sindh Krait in Pakistan and the Hump-nosed Pit Viper (*Hypnale hypnale*) in Sri Lanka. The imported Indian antivenoms, being non-specific, might have limited effectiveness in treating the envenoming caused by these endemic species.

The venoms of Big Four snakes exhibit intra-species venom variation across distant geographical areas, e.g., from various locales in Pakistan, India, Sri Lanka, and Bangladesh [10,11,13,16,18,19]. Among these highly venomous snakes, the Saw-scaled Vipers (*E. carinatus* spp.) is a complex group of diverse vipers constituting at least four subspecies in South Asia, and their venom compositions are anticipated to be even more variable across distant geographical locales. The Pakistani subspecies of Sochurek’s Saw-scaled Viper (*Echis carinatus sochureki*) is regarded as the deadliest snake in Pakistan [20,21,22]. As a widely distributed subspecies in Pakistan, it is responsible for most of the envenoming cases in the country, especially in the drier region of the Sindh Province [7,23,24]. The Multiscale Saw-scaled Viper (*Echis carinatus multisquamatus*) has a smaller range of distribution, mainly in the northern part of the country. Comparatively, snakebite envenoming caused by the latter subspecies is less common in Pakistan [25]. Of note, the Saw-scaled Viper venom used in the production of Indian antivenoms is from a totally different subspecies, i.e., *Echis carinatus carinatus*, typically sourced from Tamil Nadu, a state in the extreme south of the Indian subcontinent by the Irula Snake-Catchers’ Cooperative in Chennai. Hence, the lack of effectiveness of Indian antivenoms in treating Sochurek’s Saw-scaled Viper envenoming may be explained by the snake’s differences in subspecies and geographical origins that are far apart [14]. In fact, remarkable variation in Western Russell’s Viper (*D. russelii*) venom has been demonstrated between the Pakistani and Indian specimens [10,13,26]; notably, the Indian antivenoms were found not very effective in neutralizing the Pakistani Russell’s Viper venom. Therefore, local efforts were spearheaded by the Sindh Government and academic institute in 2014 to produce a domestic antivenom against Sochurek’s Saw-scaled Viper and Russell’s viper of Pakistan. The early batch of this bivalent antivenom was trialed in a small-scale (n = 6) clinical study in Pakistan, with a promising outcome that suggested the effectiveness of the domestic antivenom [27]. However, the antivenom production was slow and subject to interruption, ostensibly for technical and financial reasons in the early phase. A newer batch of the antivenom applying the same bi-species venom immunogen and a low-dose multi-site immunization protocol in horses [28,29] has been produced under a more sustainable government-funded program more recently. Hypothetically, the bivalent product, called Pakistani Viper Antivenom (PVAV), has high composition purity and species specificity for Pakistani viper venoms. This study therefore aimed to assess the physicochemical composition of PVAV through biochemical and proteomic approaches and to examine the immuno-specificity of the antivenom toward the venoms of various medically important snakes in the region. The study further examined the neutralization efficacy of PVAV against the venom toxicity of the three Pakistani vipers of medical importance, i.e., *E. carinatus sochureki* and *D. russelii* whose venoms are used to raise the antivenom, as well as the closely related *E. carinatus multisquamatus*.

## 2. Results

### 2.1. Chromatographic and Electrophoretic Profiling of Pakistani Viper Antivenom (PVAV)

**Figure 1:**
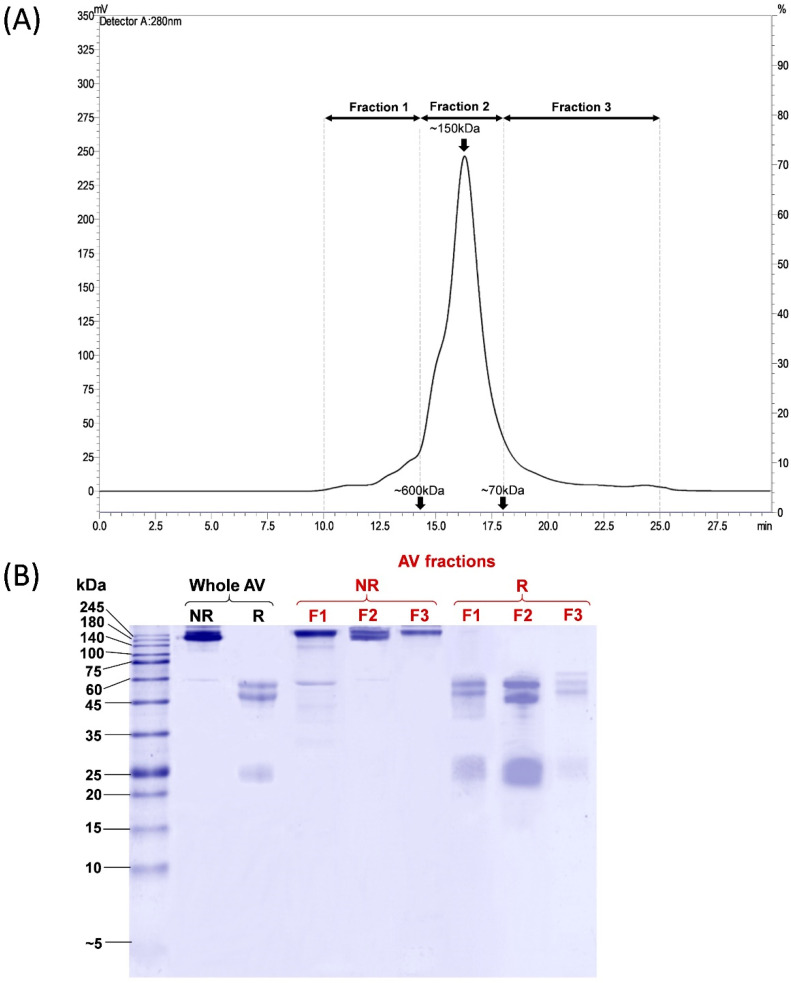
Chromatographic and electrophoretic profiling of Pakistani Viper Antivenom (PVAV). (**A**) Size exclusion chromatography fractionation of PVAV. (**B**) 15% Sodium dodecyl sulfate-polyacrylamide gel electrophoresis of PVAV under reducing and non-reducing conditions. Antivenom fractions were denoted as F1, F2 and F3. Abbreviation: AV, antivenom; NR, non-reducing; R, reducing.

PVAV was resolved by size-exclusion chromatography (SEC), and the eluents were manually collected into three fractions, namely Fractions 1, 2, and 3, corresponding to proteins of different molecular masses (Figure 1A). Most proteins were eluted in Fraction 2, accounting for ~81.9% of the total antivenom proteins based on the chromatogram peak area under the curve (AUC). Based on the standard calibration, proteins in this fraction have a molecular mass of ~150 kDa. Proteins in Fraction 1 and Fraction 3 constituted the remaining antivenom proteins, with a relative abundance of 7.7% and 10.4%, respectively (Figure 1A). 

The electrophoretic profiles of the whole Pakistani Viper Antivenom (PVAV) and its fractions (Fraction 1 to 3) under non-reducing and reducing conditions are shown in Figure 1B. The protein bands of PVAV were distributed mainly in the region with a molecular mass of ≥150 kDa under non-reducing conditions. The antivenom proteins were further reduced and separated into two major protein bands with a molecular mass of 50–60 kDa and ~25 kDa, respectively. SDS-PAGE of the antivenom fractions under non-reducing conditions yielded similar profiles with protein bands in the region of ≥150 kDa. Under reducing conditions, the majority of proteins in F1–F3 were reduced into bands of 50–60 kDa and ~25 kDa, similar to that observed in the reducing SDS-PAGE of PVAV (Figure 1B).

### 2.2. Protein Concentration and Liquid Chromatography-Tandem Mass Spectrometry (LCMS/MS) of Pakistani Viper Antivenom (PVAV) for Protein Identification

Bicinchoninic acid (BCA) assay showed that PVAV has a protein concentration of 38.4 ± 2.4 mg/mL (approximated to 3.8 g/dL or 3.8% *w*/*v*). The calibration data for protein concentration determination are provided as supplementary information in Figure S1. The identities and relative abundances of antivenom proteins in the respective SEC fractions are shown in Table 1.

**Figure 2:**
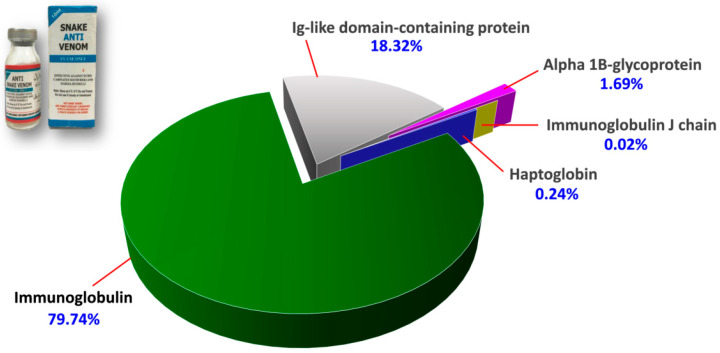
Proteome of Pakistani Viper Antivenom (PVAV, inset). The percentage values indicate the relative abundances of proteins in the antivenom.

The protein scores, mass spectral data (intensities, masses and charges of ions) and amino acid sequences of the proteins identified were provided in Supplementary File S1. Figure 2 illustrates the proteome assembled for PVAV, where immunoglobulin proteins were shown to be the most abundant component in the antivenom, constituting 79.74% of the total proteins. This is followed by Ig-like domain-containing protein (18.32%) and other minor proteins present at a low abundance level (collectively < 2%). These minor proteins include alpha-1B-glycoprotein (1.69%), haptoglobin (0.24%) and immunoglobulin J chain (0.02%) (Figure 2).

### 2.3. Immuno-Specificity of Pakistani Viper Antivenom (PVAV) 

**Figure 3:**
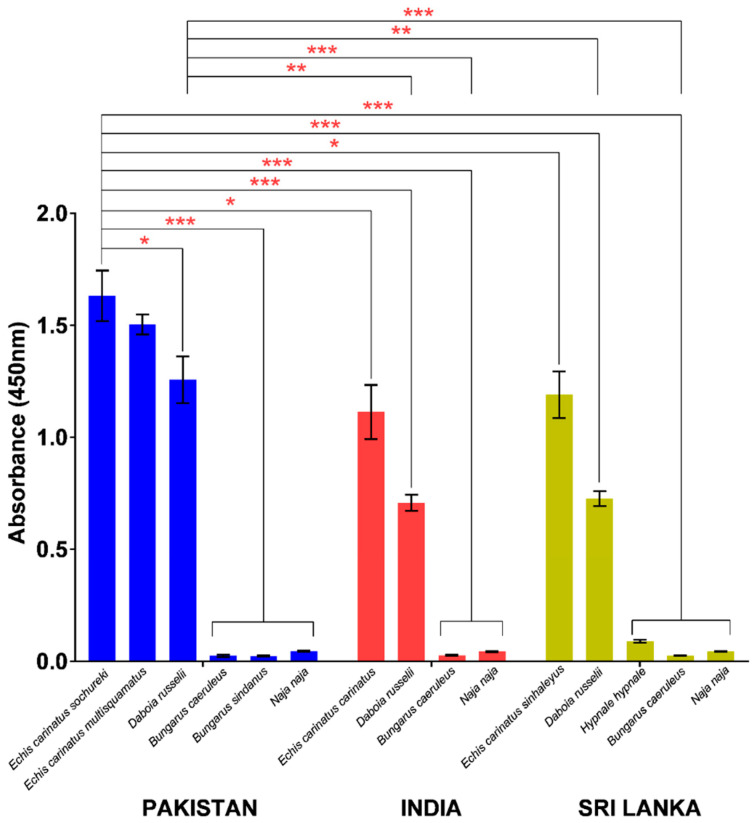
Immunological binding activities of Pakistani Viper Antivenom (PVAV) toward medically important snakes of Pakistan, India, and Sri Lanka. * Significant difference (*p* < 0.05); ** Significant difference (*p* < 0.01); *** Significant difference (*p* < 0.001).

In indirect ELISA, PVAV showed high immunoreactivity toward the venoms of *E. carinatus sochureki*, *E. carinatus multisquamatus*, and *D. russelii* from Pakistan (Figure 3). The PVAV immunoreactivity was not significantly different between the Pakistani *E. carinatus sochureki* and *E. carinatus multisquamatus* venoms (absorbance values, Abs = 1.63 ± 0.11 and 1.50 ± 0.04, respectively; *p* > 0.05). The antivenom binding activity was lower in the Pakistani *D. russelii* venom (Abs = 1.26 ± 0.10) compared with that of *E. carinatus sochureki* (*p* < 0.05), but not significantly different from the venom of *E. carinatus multisquamatus* (*p* > 0.05). The antivenom showed extremely low or negligible immunoreactivity (Abs < 0.05) toward the hetero-specific venoms of other Pakistani elapids, i.e., *N. naja*, *B. caeruleus*, and *B. sindanus* (Figure 3). 

PVAV demonstrated high immunoreactivity toward venoms of other Saw-scaled Viper subspecies from southern India (*E. carinatus carinatus*) and Sri Lanka (*E. carinatus sinhaleyus*) as well as Russell’s Vipers from the two distant regions. Its immunoreactivities were comparable between *E. carinatus carinatus* (India) and *E. carinatus sinhaleyus* (Sri Lanka) venoms (Abs of 1.11 ± 0.12 and 1.19 ± 0.10, respectively) but significantly lower in comparison to its immunoreactivity toward the venom of *E. carinatus sochureki* from Pakistan (Abs = 1.63 ± 0.11; *p* < 0.05). A similar trend of immunoreactivity was observed in the Russell’s Viper venoms, where the PVAV immunoreactivity toward the Pakistani venom specimen (Abs = 1.26 ± 0.10) was significantly higher in comparison to the venom specimens from India (Abs = 0.71 ± 0.04) and Sri Lanka (Abs = 0.73 ± 0.03) (*p* < 0.01). The antivenom also showed low to negligible immunoreactivity toward the venoms of *N. naja* and *B. caeruleus* from the two distant regions. In addition, its immunoreactivity toward the Sri Lankan Hump-nosed Pit Viper (*H. hypnale*) venom was extremely low (Abs = 0.09 ± 0.001) when compared with the Pakistani viper venoms (*E. carinatus sochureki* and *D. russelii*) (*p* < 0.001) (Figure 3). 

### 2.4. Procoagulant Activity of Pakistani Viper Venoms and Neutralization by Antivenom

The venoms of Pakistani Saw-scaled Vipers (*E. carinatus sochureki* and *E. carinatus multisquamatus*) and Russell’s Viper (*D. russelii*) exhibited potent procoagulant activity on citrated human plasma (Table 2). Among the three venoms, *D. russelii* venom showed a significantly higher procoagulant activity, with a minimum clotting dose (MCD) of 0.003 µg/mL. This value indicates a venom procoagulant effect that is at least ~11-fold more potent than the venoms of *E. carinatus multisquamatus* (MCD = 0.04 µg/mL) and *E. carinatus sochureki* (MCD = 0.10 µg/mL) (Table 2). Of the two subspecies of Saw-scaled vipers, *E. carinatus multisquamatus* venom was significantly more potent than *E. carinatus sochureki* venom. In the neutralization study, PVAV effectively neutralized the venom procoagulant effects of the three viper species, with the lowest efficacy against *D. russelii* venom (effective dose, ED = 0.02 µL), followed by *E. carinatus multisquamatus* (ED = 0.09 µL) and *E. carinatus sochureki* venoms (ED = 0.23 µL) (Table 2). 

### 2.5. Hemorrhagic Activity of Pakistani Viper Venoms and Neutralization by Antivenom

In the hemorrhagic assay, the Pakistani *E. carinatus sochureki* and *E. carinatus multisquamatus* venoms induced dermal hemorrhages in mice, both with a minimum hemorrhagic dose (MHD) of approximately 27.0 µg and a minimum hemorrhagic index (MHI) of ~870.0–883.0 unit (*p* > 0.05) (Table 3). On the other hand, the *D. russelii* venom showed negligible dermal hemorrhagic activity. In the neutralization study, PVAV was significantly more efficacious in neutralizing the hemorrhagic effect of *E. carinatus multisquamatus* venom (ED_50_ = 7.9 µL) than *E. carinatus sochureki* venom (ED_50_ = 16.6 µL) (*p* < 0.01) (Table 3).

### 2.6. Venom-Induced Lethality of Pakistani Viper Venoms and Neutralization by Antivenom

In mice, *E. carinatus sochureki* venom has an intravenous median lethal dose (LD_50_) of 2.57 µg/g, which is higher than that of *E. carinatus multisquamatus* venom (1.57 µg/g) (Table 4). The venom lethality of both Saw-scaled Vipers was lower than Russell’s Viper venom, whose LD_50_ was previously reported to be 0.19 µg/g in mice for the same venom stock [10]. In the neutralization study, PVAV effectively neutralized the lethality of all venoms. It has the highest efficacy against the *E. carinatus sochureki* venom with a potency (P) of 21.59 mg/mL, which is equivalent to complete neutralization of 0.40 LD_50_ per microliter of PVAV. The antivenom was less efficacious in neutralizing the lethality of *D. russelii* venom and *E. carinatus multisquamatus* venoms (P = 1.13 mg/mL and 3.50 mg/mL, respectively). In terms of the number of LD_50_ being neutralized per microliter of antivenom, PVAV was approximately 2.5-fold more potent against *D. russelii* venom (P = 0.28 LD_50_/µL) than against the non-immunizing *E. carinatus multisquamatus* venom (P = 0.11 LD_50_/µL) (Table 4). In addition, the normalized potency (n-P) values were expressed based on antivenom protein concentration for comparison purposes between different antivenom products (see also Table S1 and discussion in Section 3).

## 3. Discussion

The chromatographic profile of PVAV showed that the main protein component (81.9% based on AUC) in the antivenom is approximately 150 kDa, consistent with immunoglobulin G (IgG). In SDS-PAGE, the protein was separated under reducing conditions into two major bands with molecular weights of 50–60 kDa and ~25 kDa, which are indicative of the intact heavy chain and light chain of IgG, respectively. The heavy chain of IgG in PVAV is an intact molecule in which the Fab portion and Fc fragment remain covalently bonded, unlike F(ab’)_2_ antivenom whose Fc fragment has been proteolytically removed, leaving behind a cleaved heavy chain with a molecular weight of ~28 kDa [30,31]. In SDS-PAGE, the heavy chain appeared as multiple bands closely migrated in the region of ~50–60 kDa under reducing conditions. These could be due to the unfolding of the CH2, CH3, or variable domain in the “Y” structure of the IgG molecule caused by the heating process during sample preparation [32]. Another possible cause of closely migrated bands in the ~50–60 kDa region may be the presence of different IgG isotypes in the antivenom product [33]. In this study, the analysis with liquid chromatography and tandem mass spectrometry (LCMS/MS) further verified that immunoglobulins constitute the major components of PVAV.

Accordingly, PVAV as a whole IgG antivenom product may have several advantages over the preparations of F(ab’)_2_ or Fab antivenom. The IgG molecule has a longer elimination half-life and a smaller volume of distribution compared with the F(ab’)_2_ and Fab molecules (F(ab’)_2_ is intermediate) [34]. Its long elimination half-life (~60 h) indicates that the IgG antivenom has a low clearance from the body, permitting a longer therapeutic action of neutralization against the venom toxicity. This also reduces the likelihood of recurrent envenoming, which may result from a pharmacokinetic and pharmacodynamic mismatch between venom and antivenom, as seen in envenomed patients treated with Fab or F(ab’)_2_ antivenoms [35,36,37]. The longer half-life and smaller volume of distribution of IgG antivenoms, such as PVAV, imply that the antibodies distribute mainly in the vascular compartment, thus allowing the antivenom to bind and neutralize the viperid hemotoxins more readily [34,38]. As an intact IgG molecule, it carries the Fc fragment, which has been suggested to be allergenic [39,40,41]. Nonetheless, several clinical studies showed the use of IgG antivenom is unlikely associated with an increased risk of hypersensitivity reactions [41,42,43]. More importantly, the impurities or the presence of non-therapeutic proteins in antivenom should be addressed, as these are known allergenic components commonly found in many products [9,40]. These protein impurities in antivenom products are not typically noticeable based on chromatography or SDS-PAGE alone due to their low abundances or being masked by immunoglobulin fragments during chromatographic or electrophoretic profiling. 

Therefore, this study further explored the protein composition details of PVAV by proteomics. LCMS/MS analysis confirmed the presence of equine immunoglobulins that made up ~80% of the total antivenom proteins; this is an acceptable high level of immunoglobulin compared to many antivenom products that are clinically used for snakebite treatment [30,31,44,45]. The proteomic analysis also detected Ig-like domain-containing protein (18.32%) consisting of sequence structure of equine immunoglobulin domain known for antigen-binding activity [46,47]. Other minor proteins not apparent on chromatography and SDS-PAGE were detected along with LCMS/MS. These were mainly non-immunoglobulin G serum proteins, such as alpha-1B-glycoprotein, haptoglobin, and immunoglobulin J chain (collectively < 2% of total antivenom proteins). Notably, a common protein contaminant, i.e., serum albumin, was not detected, suggesting a stringent and effective purification process applied in the production of PVAV. Based on the WHO guideline on antivenom production, the albumin content in an antivenom product should not exceed 1% of the total antivenom proteins [9]. The presence of protein impurities, typically non-IgG serum proteins, is usually because of the imperfect purification process of IgG antibodies during antivenom production. The most common practice for antibody purification in antivenom production nowadays is by protein precipitation method using ammonium sulfate or caprylic acid, which results in different recovery rates of the antibodies (40–50% and 60–75%, respectively) [48]. It has been suggested that in antivenom production, higher purity of immunoglobulin proteins can be achieved with the caprylic acid precipitation method [9,49,50,51,52]. In this context, PVAV is purified using the caprylic acid precipitation method, and this may explain the relatively high content purity of the antivenom. Furthermore, the protein concentration of PVAV (38.4 mg/mL or 3.84 g/dL) is well within the acceptable range as per the WHO guideline, which recommends that the total protein concentration in an antivenom should not exceed 10 g/dL [9] in order to minimize the risk of hypersensitivity induced by animal proteins. 

Envenoming inflicted by Saw-scaled Vipers (*Echis* spp.) and Western Russell’s Vipers (*D. russelii*) commonly result in venom-induced consumptive coagulopathy (VICC) [53,54,55]. The present study showed Pakistani *E. carinatus* subspecies (*E. carinatus sochureki* and *E. carinatus multisquamatus*), and *D. russelii* venoms exhibited very potent procoagulant activity on human plasma (MCD ≤ 0.1 µg/mL), consistent with the coagulopathic effect of envenoming caused by these species. The potent procoagulant effects of these venoms are likely induced by snake venom metalloproteinases (SVMPs) present in the venoms as in the forms of factor X activating enzyme for *D. russelii* [10,56], and EC-PIII, carinactivase [57], ecarin [58] as well as other prothrombin activating SVMPs for *Echis carinatus* [59,60]. Meanwhile, the venoms of Pakistani *Echis* vipers but not *D. russelii* induced dermal hemorrhagic effect in mice. The hemorrhagic venom activities of the *Echis* vipers are contributed by the high abundances of hemorrhagic SVMPs (~20–54%) in the venoms [61,62,63]. In contrast, the Pakistani *D. russelii* venom contains an exceptionally low abundance of SVMP (~2.5%) [10], consistent with the lack of local hemorrhagic effect of the venom, as seen in this study. Nonetheless, the Pakistani *D. russelii* is extremely lethal with a low LD_50_ of 0.19 µg/g, a value comparable to those of many neurotoxic elapid venoms (LD_50_ < 0.2 µg/g) [64,65]. The high lethal potency of Pakistani *D. russelii* venom is likely attributed to the high abundance of toxic PLA_2_s, which constitute ~33–64% of total venom proteins based on quantitative proteomics [10,13,66]. The PLA_2_s of Russell’s Viper venom are known to exhibit a wide spectrum of toxicity, including neurotoxicity for the Sri Lankan species [67], nephrotoxicity for the Burmese species [68,69], and invariably anticoagulant activity responsible for coagulopathic envenoming in most cases of Russell’s viper bite [70,71,72]. On the other hand, the venoms of *E. carinatus sochureki* and *E. carinatus multisquamatus* are less lethal than the *D. russelii* venom, consistent with their lower coagulotoxic effects observed *in vitro*. 

Snake venom variation due to geographical factors is common within the same species, as exemplified by many proteomic studies on the venoms of *Echis* [18,62] and *Daboia* vipers [11,13,66]. Consequently, the choice of venom immunogen during antivenom production is crucial to raise antibodies that can effectively neutralize the toxins of targeted snake species from specific areas where the envenoming is prevalent. Unlike the manufacturing of Indian polyvalent antivenoms, the immunogen of PVAV incorporates the venoms of the two most medically important native vipers in Pakistan, i.e., *E. carinatus sochureki* and *D. russelii*, while excluding the venoms of elapid snakes (cobra and krait) from the immunogen formula. The hyperimmunization with this “Pakistani viperids only” venom immunogen should result in a higher antibody titer with good avidity toward the Pakistani viperid toxins. The antivenom produced thereof should have high specific immunoreactivity and neutralization efficacy against these indigenous species whose venoms are used as the immunogen. Indeed, PVAV was found to be highly immuno-specific to venoms of the Pakistani vipers (*E. carinatus sochureki* and *D. russelii*) and efficacious in neutralizing the principal toxicities (procoagulant, hemorrhagic, and lethal effects) caused by the venoms. The finding suggests that the immunization process is effective in raising a Pakistani viper-specific antivenom which can be used in Pakistan for hemotoxic envenoming. Moreover, although *E. carinatus multisquamatus* venom was not included as part of the immunogen, PVAV exhibited substantially high cross-reactivity and para-specific neutralization activity against this venom, further supporting its utility for viperid envenoming in the country.

Comparing the venoms of Pakistani Saw-scaled Vipers and Russell’s Viper, PVAV immunoreactivity and neutralizing efficacy were apparently lower toward the latter, implying the *D. russelii* venom is less immunogenic or has a lower protein antigenicity for immunorecognition by the bivalent PVAV. Previous venom proteomics of the two Pakistani vipers showed their venom compositions are substantially varied. The major proteins in Saw-scaled Viper venom are snake venom metalloproteinases (SVMPs) (54.7%) [63], whereas the Pakistani *D. russelii* has a very high abundance of phospholipases A_2_ (PLA_2_) (63.8%) [10]. In general, the immunogenicity of toxin is influenced by the protein molecular weight: the larger the protein molecule (antigen) is, the more immunogenic it is [73]. SVMPs generally have a much higher molecular weight ranging from 30–100 kDa (depending on the subclass, with P-III SVMP being the largest due to the presence of multiple domains and occasionally complex formation) [74,75,76], while PLA_2_ has a relatively lower molecular weight of ~13–14 kDa [77,78,79]. Hence, PVAV’s higher immunoreactivity toward the Saw-scaled Viper venoms compared with *D. russelii* venom might be due to the presence of higher antibody titers toward the SVMPs. 

The definitive treatment of snakebite envenoming is the intravenous injection of specific antivenom [1]. However, in most under-developed and developing countries, such as Pakistan, effective antivenoms are limited or non-existent for the treatment of snakebite envenoming. For decades, Pakistan relies heavily on imported antivenoms from other countries especially India to treat snakebite envenoming cases, although questions have been frequently raised regarding their lack of effectiveness when applied in the Pakistani setting against the envenoming by Pakistani venomous snake species [14,80]. To further evaluate the applicability of various antivenom products against Saw-scaled viper and Russell’s viper envenoming in Pakistan, recent antivenom assessment studies (including present work) were reviewed and compared as provided in Table S1. Three foreign products, i.e., VPAV and Premium Serums produced in India, and ICP produced in Costa Rica, were raised against a mix of four snake venoms (Big Four venoms, except in the case of ICP where *B. caeruleus* venom was excluded while hump-nosed pit viper venom was added). While all four antivenoms (PVAV, VPAV, ICP, Premium Serums) have been tested independently against the venom lethality of Pakistani Russell’s Viper (notwithstanding different venom stock), only PVAV from the current work was used to neutralize the two Pakistani Saw-scaled viper venoms. Efficacy comparison was therefore done on the neutralization of Russell’s Viper venom based on normalized potency, in view of the varying protein concentrations among the four antivenom products (Table S1). In comparison, PVAV has the highest normalized potency (29.43 mg/g, amount of venom completely neutralized per one gram of antivenom proteins) as shown in the current study, and this was followed closely by the Premium Serums product (n-P = 28.34 mg/g) derived from Pla et al. [13]. ICP and VPAV were found to be slightly less efficacious (n-P = 25.62 mg/g and 22.32 mg/g, respectively) by Pla et al. [13] against the same Pakistani Russell’s Viper venom used in the study [13]. Interestingly, another study using a different sample of Pakistani *D. russelii* venom showed a markedly low efficacy of the Indian polyvalent antivenom (n-P = 2.70 mg/g) [10], implying potential venom variability even within the population of Pakistani Russell’s Vipers. In comparison, PVAV outperformed the imported antivenoms, underscoring the need for locale-defined and species-specific antivenom product in Pakistan. Since the Indian and Sri Lankan Saw-scaled Vipers are completely of subspecies different from those in Pakistan, further study is important to verify if the foreign antivenoms also lack efficacy against the Pakistani *Echis* venoms. 

As PVAV is regarded as a newly developed domestic antivenom product, the present study also explored its immunoreactivity toward venoms of various snake species in the region. While PVAV showed negligible immunoreactivity toward the elapid venoms (of cobra and krait) from different geographical locales of South Asia, it was found to be immunoreactive toward the venoms of closely related *E. carinatus* subspecies, which are *E. carinatus carinatus* from southern India and *E. carinatus sinhaleyus* from Sri Lanka, as well as Russell’s Viper venoms from the two distant regions, albeit its immunoreactivity was considerably lower (~50% less) toward the venoms of these vipers of non-Pakistani origins. Of note, the antivenom immunoreactivities toward the two Saw-scaled Vipers from southern India and Sri Lanka were comparable, implying that the venom antigenic properties of the two subspecies of *E. carinatus* are considerably conserved. The same was observed when comparing Russell’s Vipers from southern India and Sri Lanka, PVAV immunoreactivities were not significantly different between the two venom specimens. The observation suggests that the snakes (per species) from southern India (Tamil Nadu) and Sri Lanka likely have venoms that are less distinct antigenically. Presumably, the vipers from both locales share geographical and evolutionary proximity, and habitats with similar climates, hence the conserved venom phenotype which has also been demonstrated proteomically [11].

As observed between the Pakistani Saw-scaled Viper and Russell’s Viper venoms, PVAV’s higher immunoreactivity toward the former was also shown in venom samples from southern India and Sri Lanka. The consistently higher immunoreactivity of PVAV toward Saw-scaled Viper venom compared with *D. russelii* venom could be due to a higher antibody titer toward the high MW metalloproteinases, which are present more abundantly in Saw-scaled Viper venom. In addition, the lack of PVAV cross-reactivity toward the Sri Lankan Hump-nosed Pit Viper (*H. hypnale*) venom implies that this crotaline venom antigenicity is substantially varied from that of true vipers (Saw-scaled Vipers and Russell’s Viper), despite having toxins protein families that are common in Viperidae snake venoms [81]. Together, the high immuno-specificity of PVAV corroborates its immunoreactivity and neutralizing activities for Pakistani Saw-scaled Vipers and Russell’s Viper venoms. Its moderate immunoreactivity toward the venoms of other Saw-scaled Viper subspecies and Russell’s Viper from southern India and Sri Lanka suggests its potential geographical utility in the region.

Furthermore, the formulation of PVAV as an anti-viperid bivalent antivenom implies the feasibility of streamlining antivenom production into two different types of antivenom, one for viperids and one for elapids, the use of which can be indicated based on a syndromic approach for snakebite envenoming in the region. Ideally, monovalent (mono-specific) antivenom is the choice of treatment since it should contain a higher portion of antibodies specific to the venom of a particular snake species [1]. Unfortunately, species diagnosis is usually not easy clinically as doctors in general are not well trained for snake species identification. Nevertheless, in Pakistan, the hemotoxic and neurotoxic syndromes caused by viperids or elapids are clinically distinguishable, thus favoring the use of dichotomous syndrome-based antivenom products as exemplified by PVAV in this study. 

## 4. Conclusions

The study shows that the bivalent Pakistani Viper Antivenom (PVAV) is an immunoglobulin G (IgG) antivenom product with relatively high physicochemical purity. Based on antivenom proteomics, PVAV is composed of primarily immunoglobulins with little impurities, notably the absence of serum albumin. PVAV is highly immuno-specific toward the venoms of immunizing viper species, i.e., *E. carinatus sochureki* and *D. russelii* from Pakistan, as well as the non-immunizing indigenous Pakistani *E. carinatus multisquamatus* venom. The findings of toxicity neutralization further suggest PVAV is potentially a useful domestic antivenom product for the treatment of viperid envenoming in Pakistan. Clinical trials are warranted to verify the product’s effectiveness and superiority over the imported foreign antivenoms. 

## 5. Materials and Methods

### 5.1. Antivenoms and Venoms

The Pakistani venoms of *E. carinatus sochureki*, *E. carinatus multisquamatus*, *D. russelii*, *B. sindanus*, *N. naja*, and *B. caeruleus* were obtained from the Anti-Snake Venom (ASV)/Anti-Rabies (ARV) Serology Laboratory, Pakistan. Indian snake venoms of *E. carinatus carinatus*, *D. russelii*, *B. caeruleus*, and *N. naja* were supplied by VINS Bioproduct Limited, India. Sri Lankan snake venoms of *Echis carinatus sinhaleyus*, *D. russelii*, *H. hypnale*, *B. caeruleus*, and *N. naja* were sourced from the research serpentarium of the University of Colombo, Sri Lanka. All venoms were collected from a minimum of 5 adult snakes for each species, lyophilized, and kept at −20 °C until further use.

The bivalent Pakistani Viper Antivenom (PVAV) used in the present study is a liquid antivenom product supplied by the ASV/ARV Serology Laboratory, Peoples University of Medical and Health Sciences for Women Shaheed Benazirabad, Sindh, Pakistan. PVAV is developed from sera of horses hyperimmunized against the venoms of Sochurek’s Saw-scaled Viper (*E. carinatus sochureki*) and Russell’s Viper (*D. russelii*) from Pakistan.

### 5.2. Animal

Albino mice of Institute of Cancer Research (ICR strain, 20–30 g) were supplied by the Animal Experimental Unit (AEU), Faculty of Medicine, University of Malaya. The animals were handled according to the Council for International Organization of Medical Sciences (CIOMS) guideline on animal experimentation [82]. All methods were carried out in accordance with the guidelines and regulations approved by the Institutional Animal Care and Use Committee (IACUC) of University of Malaya (Protocol approval number: 2021-220506/PHAR/R/TCH).

### 5.3. Estimation of Antivenom Protein Concentration

Protein concentrations of antivenom (Pakistani Viper Antivenom, PVAV) were determined using Thermo Scientific™ Pierce™ BCA (bicinchoninic acid) protein assay kit (Rockford, IL, USA) with bovine serum albumin (BSA) as protein standard calibration (concentration range: 0–10 mg/mL). The protein concentrations were expressed as means ± standard error of the mean (S.E.M.) of triplicates.

### 5.4. Size-Exclusion Chromatography 

Size-exclusion chromatographic (SEC) fractionation of Pakistani Viper Antivenom (PVAV) was performed using a Yarra 3 μm SEC-3000, 300 × 7.8 mm size-exclusion column (Phenomenex, Torrance, CA, USA). Two milligrams in the total volume of 200 µL of PVAV were injected into the column. The elution buffer used was a mixture of 100 mM sodium phosphate buffer (pH 6.8), and 300 mM sodium chloride, eluted at a flow rate of 0.5 mL/min. Proteins in the antivenom were detected by absorbance readings at 280 nm for 30 min. Eluted PVAV protein fractions were manually collected in 2 mL centrifuge tubes consecutively accordingly over the elution time of each fraction. Collected fractions were then concentrated and desalted using Sartorius Vivaspin^®^ 20 concentrators (Goettingen, Germany) and stored at 4 °C until further use. Calibration of the column was done using the following protein standards supplied by Supelco^®^ Sigma-Aldrich (Darmstadt, Germany): thyroglobulin bovine (670 kDa), γ-globulin (150 kDa), albumin chicken egg grade VI (44.3 kDa), ribonuclease A type I-A from bovine pancreas (13.7 kDa), and p-aminobenzoic acid (PABA) (0.137 kDa).

### 5.5. In-Solution Tryptic Protein Digestion and Liquid Chromatography-Tandem Mass Spectrometry (LCMS/MS) 

Protein fractions collected from the size-exclusion chromatography (SEC) were reduced by dithiothreitol (DTT) (Sigma-Aldrich, Saint Louis, MO, USA), alkylation by iodoacetamide (Sigma-Aldrich, Saint Louis, MO, USA), and then digested by mass-spectrometry grade trypsin protease as previously described [83]. The trypsin-digested peptides were desalted with Millipore ZipTip^®^ C18 Pipette Tips (Merck, NJ, USA). In brief, trypsin-digested peptides were dissolved by adding 7 μL of 0.1% formic acid in water and subjected to nano-electrospray ionization liquid chromatography-tandem mass spectrometry (ESI-LCMS/MS) through the Agilent 1200 HPLC-Chip/MS Interface (Agilent Technologies, Santa Clara, CA, USA) coupled with Agilent 6550 iFunnel Q-TOF LC/MS. The peptide samples were loaded onto a C_18_ enrichment column (Pore size: 300 Å, 160 nL), followed by a 75 µm × 150 mm analytical column ((Agilent part No. G4240-62010). Injection volume was tuned to 1 μL per sample, and the sample elution flow rate was adjusted at 4 μL/min, with linear gradient (5–50% B for 11 min, 50–70% B for 4 min and 70% B for 3 min) of 5–75% of solvent B (0.1% formic acid in 100% acetonitrile). Drying gas flow and gas temperatures were 5 L/min and 325 °C, respectively. The fragmentor voltage was adjusted to 360 V; capillary voltage was set at 1900 V. Positive ionization mode was selected for ion polarity. Mass spectra were acquired using Mass Hunter acquisition software in an MS/MS mode with a MS scan range of 110–3000 m/z and MS/MS scan range of 50–3000 m/z. Precursor charge selection was set as doubly charge state, and above with the exclusion of precursors 299.2944 (z = 1) and 1221.9906 m/z (z = 1) set as reference ions. Data extraction was conducted with MH+ mass range between 50 and 3200 and processed with Agilent Spectrum Mill MS Proteomics Workbench software package version B.06.00.201. The carbamidomethylation of cysteine residues was set as a fixed modification and oxidation of methionine residues as variable modification. The resulting raw mass spectra were searched against NCBI non-redundant Equidae database (taxid: 9788). The proteins in each antivenom fraction were then identified based on the similarity matches of peptides. Protein identifications were validated and adjusted as previously described [30] with the following filters: protein score > 10, peptide score > 10, and scored peak intensity (SPI) > 60%. Results with “Distinct Peptide” identification of ≥2 will be considered significant. Identified proteins were filtered to achieve a false discovery rate (FDR) < 1% for the peptide-spectrum matches.

The relative protein abundances were estimated based on the chromatographic peak area under the curve (AUC) and the ratio of mean spectral intensity (MSI) of protein relative to the total MSI of all proteins identified [84], calculated using the formula as follows:

(1) $$\begin{array}{c} \mathrm{Relative}\;\mathrm{abundance}\;\mathrm{of}\;\mathrm{Protein}\;X\;\mathrm{in}\;\mathrm{fraction}\;Y\;(\%)\;\\ =\frac{\mathrm{MSI}\;\mathrm{of}\;\mathrm{protein}\;X\;\mathrm{in}\;\mathrm{HPLC}\;\mathrm{fraction}\;Y\;}{\mathrm{Total}\;\mathrm{MSI}\;\mathrm{of}\;\mathrm{all}\;\mathrm{proteins}\;\mathrm{in}\;\mathrm{HPLC}\;\mathrm{fraction}\;Y\;}\\ \times \;\mathrm{Area}\;\mathrm{under}\;\mathrm{the}\;\mathrm{curve}\;(\mathrm{AUC})\;\mathrm{of}\;\mathrm{HPLC}\;\mathrm{fraction}\;Y\;(\%) \end{array}$$

The MSI of protein X in fraction Y refers to the mean spectral intensity of peptides ion assigned to protein X eluted in HPLC fraction Y. The AUC of fraction was determined from the chromatogram using the Shimadzu LC solution Software (Version 1.23, Shimadzu, Kyoto, Japan, 2007).

### 5.6. Sodium Dodecyl Sulfate-Polyacrylamide Gel Electrophoresis (SDS-PAGE)

Electrophoretic profiling of Pakistani Viper Antivenom (PVAV) was carried out based on the method described previously [85]. In brief, 5 µg protein of the antivenom and SEC-fractionated antivenoms (F1 to F3) were reconstituted in ultrapure water and mixed with loading buffer in a 1:5 volume ratio, boiled for 10 min, and loaded into discontinuous reducing and non-reducing 15% polyacrylamide gel. Electrophoresis was conducted for 2 h at 90 V. Calibration was done using PM2700 ExcelBandTM 3-color Broad Range Protein Marker (5–245 kDa) (SMOBIO Technology Inc., Taiwan). The gel’s protein bands were visualized through staining with Coomassie Brilliant Blue R-250 and observed using Image Scanner III Labscan 6.0 (GE Healthcare, Freiburg, Germany) as described previously [86]. 

### 5.7. Indirect Enzyme-Linked Immunosorbent Assay (ELISA)

Immunological binding activities between antivenom (PVAV) and venoms (*E. carinatus sochureki*, *E. carinatus multisquamatus*, *E. carinatus carinatus*, *E. carinatus sinhaleyus*, *D. russelii*, *Bungarus sindanus*, *Bungarus caeruleus*, and *H. hypnale*) were examined with an indirect enzyme-linked immunosorbent assay (ELISA) modified from Tan et al. [31]. In brief, 96-well immunoplate (SPL Lifesciences, Pocheon-si, Korea) were pre-coated overnight with 10 ng venoms in 100 µL carbonate-bicarbonate coating buffer at 4 °C. The plate was flicked dry and rinsed with phosphate-buffered saline containing 0.5% Tween^®^20 (PBST) four times. Antivenoms were prepared in a stock concentration of 20 mg/mL, and appropriately diluted antivenom (1:2700) was added to each venom-coated well and incubated at room temperature for one hour, and similarly washed with PBST after the incubation. Appropriately diluted horseradish peroxidase-conjugated anti-horse-IgG (Jackson ImmunoResearch Inc., West Grove, PA, USA) in PBST (1:10,000) was added to the well and incubated for 1 h at room temperature. Another cycle of washing steps with PBST was done to remove excess components, and 50 µL of a substrate solution (3,3′,5,5′-Tetramethylbenzidine, Elabscience, TX, USA) was added to each well. The enzymatic reaction was allowed in the dark for 30 min at room temperature and subsequently terminated by adding 50 µL of 12.5% sulfuric acid. The absorbance (Abs) was read against the blank using a SpectraMax^®^ ABS Plus microplate reader (Molecular Devices, San Jose, CA, USA) at 450 nm. Immunological binding activity was expressed as relative absorbance. Values were presented as means ± standard error of the mean (S.E.M.) of triplicate experiments. Values were presented as means ± standard error of the mean (SEM) of triplicate experiments. One-way analysis of variance (ANOVA) with Tukey’s Honest Significant Difference (HSD) test was used to examine the statistical significance.

### 5.8. Procoagulant Activity of Venoms and Neutralization by Antivenoms 

The procoagulant activities of venoms were examined using citrated human plasma. 100 µL venom samples with various dilutions in saline were loaded into 96-microplate wells at 37 °C. 100 µL Citrated human plasma containing 0.4 M CaCl_2_ was then added to each well simultaneously. The coagulation of plasma was measured immediately using a modified turbidimetric method [11,87]. Clot formations were monitored at 405 nm (absorbance) using multimode plate reader for 30 min, with readings taken at 30 s intervals. An increase of 0.02 units from the mean of the preceding absorbance measurements indicated the plasma clotting time of the venom at a specified concentration. A concentration-response curve was drawn, and the venom concentration which induced plasma coagulation in 3 min is known as the minimum clotting dose (MCD) of the venom. 

The effective dose (ED) of antivenom (PVAV) in neutralizing the procoagulant effect was defined as the dose of antivenom that prolonged the clotting time of citrated human plasma three times that of the control (2 MCD venom without antivenom). Preincubation a fixed dose of venom (2 MCD) with various doses of antivenom at 37 °C for 30 min. 100 µL Citrated human plasma with 0.4 M CaCl_2_ was added and determination of clotting time was performed as described above. Values were presented as means ± SEM of triplicate experiments. One-way analysis of variance (ANOVA) with Tukey’s Honest Significant Difference (HSD) test was used to examine the statistical significance.

### 5.9. Hemorrhagic Activity of Venoms and Neutralization by Antivenoms 

The hemorrhagic activities of venoms were examined based on the method described previously [88] with modification. Briefly, different doses of venom were prepared in a final volume of 100 µL with normal saline. The solutions were injected intradermally into the dorsal skin of ICR albino mice (n = 3 per dose, 20–25 g). The mice were euthanized by anesthetic overdose and the skins were carefully removed, exposing the dermal hemorrhagic lesions for examination after 90 min. The hemorrhagic lesion size was examined by measuring its mean diameter (in mm). Minimum hemorrhagic dose (MHD) is defined as the amount of venom (µg) that induces a skin hemorrhagic lesion of 10 mm in diameter [9]. The hemorrhagic activities of venoms at different doses were further characterized as a function of the diameter and intensity of hemorrhage and expressed as the venom hemorrhagic index (VHI) [89]. A plot of VHI against venom doses tested was created, and the VHI corresponding to the conventionally defined MHD (amount of venom in µg) was determined as the venom minimum hemorrhagic index (MHI), which serves as an indicator of potency for the venom’s hemorrhagic activity. 

In the neutralization study of hemorrhagic effect, various doses of antivenom were pre-incubated with a challenge dose of minimum hemorrhagic dose (2 MHD) of venom at 37 °C for 30 min prior to intradermal injection (n = 3 per dose). The mice were euthanized 90 min after the inoculation of venom-antivenom mixture, and the skin was removed as earlier described. The diameter (mm) and intensity of the hemorrhagic lesion that occurred at each antivenom dose were measured, and the resulting venom hemorrhagic index (VHI) was calculated as described. The neutralization of hemorrhagic effect was expressed as median effective doses (ED_50_), defined as the dose of antivenom (µL) at which the VHI in the treated mice was reduced by 50% of the control (mice which received 2 MHD without antivenom) [89]. Values were presented as means ± SEM of triplicate experiments. One-way analysis of variance (ANOVA) with Tukey’s Honest Significant Difference (HSD) test was used to examine the statistical significance.

### 5.10. Lethality of Venoms and Neutralization by Antivenom

The lethal activities of venoms were determined in ICR albino mice. The venoms at varying doses in a volume of 100 µL normal saline were injected intravenously via caudal vein into the mice (20–25 g) [90]. The mice were allowed access to food and water ad libitum. The survival ratio was recorded at 24 h, and the median lethal doses (LD_50_) of the venoms were determined with Probit analysis using BioStat 2009 analysis software (AnalystSoft Inc., Canada) [91]. In the neutralization study, a challenge dose of venom (2.5 or 5 LD_50_) was pre-incubated with various dilutions of the antivenom at 37 °C for 30 min. The mixtures were then injected intravenously into the mice, and the mice were monitored for 24 h. The median effective dose (ED_50_) of the antivenom, defined as the dose of antivenom (µL) at which 50% of mice survived against each challenge dose of venom, was determined. The venom-neutralizing capacity of antivenom was also expressed in terms of neutralization potency (denoted as ‘P’), defined by two parameters: (i) the number of LD_50_ completely neutralized per unit volume of antivenom (mL); (ii) the amount of venom (mg) completely neutralized per unit volume of antivenom (mL). The neutralization potency (P) is a direct indicator of antivenom neutralizing capacity and is theoretically unaffected by the number of challenge dose used (nLD_50_) in the neutralization study [92,93]. For comparison purposes across different antivenom products, the potency values were normalized by the respective antivenom protein concentrations and expressed in terms of normalized potency (n-P), defined as the amount of venom (mg) completely neutralized per unit amount of antivenom protein (g) [93,94].

## Figures and Tables

**Table 1:** Protein composition of Pakistani Viper Antivenom (PVAV) as fractionated by size-exclusion chromatography.

Fraction 1		Fraction 2		Fraction 3	
Protein	R.A. (%)	Protein	R.A. (%)	Protein	R.A. (%)
Immunoglobulin	7.10	Immunoglobulin	62.84	Immunoglobulin	9.80
Ig-like domain-containing protein	0.44	Ig-like domain-containing protein	17.88	Alpha-1B-glycoprotein	0.64
Immunoglobulin J chain	0.02	Alpha-1B-glycoprotein	1.00		
Alpha-1B-glycoprotein	0.05	Haptoglobin	0.18		
Haptoglobin	0.06				
Total	7.7		81.9		10.4

R.A.: Relative abundance.

**Table 2:** Procoagulant activity of Pakistani vipers (*Echis carinatus sochureki*, *Echis carinatus multisquamatus*, *Daboia russelii*) venoms and venom neutralization by Pakistani Viper Antivenom (PVAV).

Species	MCD (µg/mL) ^@^	PVAV (38.4 ± 2.4 mg/mL)
		ED (µL) ^#^
*E. carinatus sochureki*	0.1024 ± 0.0114 ^a^	0.2287 ± 0.0321 ^a^
*E. carinatus multisquamatus*	0.0409 ± 0.0032 ^b^	0.0867 ± 0.0088 ^b^
*D. russelii*	0.0034 ± 0.0007 ^c^	0.0162 ± 0.0002 ^b^

^@^ Minimum clotting dose (MCD): Minimal amount of venom (µg) required to induce clotting of 1 mL citrated human plasma in 3 min. ^#^ Effective dose (ED): A dose of antivenom (µL) is required to prolong the clotting time of 2 MCD to 3 times that of the control. Values were expressed as means ± S.E.M. Means with different superscript letters are significantly different, *p* < 0.05 (Tukey’s HSD test).

**Table 3:** The hemorrhagic activity of Pakistani vipers (*Echis carinatus sochureki*, *Echis carinatus multisquamatus*, *Daboia russelii*) venoms and venom neutralization by Pakistani Viper Antivenom (PVAV).

Species	MHD (µg) ^@^	MHI (Unit) ^#^	PVAV (38.4 ± 2.4 mg/mL)
			ED_50_ (µL) ^$^
*E. carinatus sochureki*	27.2 ± 1.2	870.0 ± 56.9	16.6 ± 0.7 ^a^
*E. carinatus multisquamatus*	26.7 ± 1.5	883.0 ± 147.1	7.9 ± 0.9 ^b^
*D. russelii*	N.A.	N.A.	N.A.

^@^ Minimum hemorrhagic dose (MHD): Dose of venom (µg) required to induce a dermal hemorrhagic lesion of 10 mm diameter. ^#^ Minimum hemorrhagic index (MHI): Venom hemorrhagic index (VHI) corresponding to the conventionally defined MHD of the venom (that induces a hemorrhagic lesion of 10 mm diameter). ^$^ Median effective dose (ED_50_): Dose of antivenom (µL) at which the venom hemorrhagic index (VHI) in the treated mice was reduced by 50% compared with the control (mice which received 2 MHD without antivenom). N.A.: Not applicable (no hemorrhagic effect)**.** Values were expressed as means ± S.E.M. Means with different superscript letters are significantly different, *p* < 0.01 (Tukey’s HSD test).

**Table 4:** Lethality of the venoms of Pakistani vipers (*Echis carinatus sochureki*, *Echis carinatus multisquamatus*, *Daboia russelii*) and venom neutralization by Pakistani Viper Antivenom (PVAV).

Species	LD_50_ (µg/g) ^a^	Challenge Dose (nLD_50_)	PVAV (38.4 ± 2.4 mg/mL)				
			ED_50_ (µL) ^b^	ER_50_ (mg/mL) ^c^	P (LD_50_/mL) ^d^	P (mg/mL) ^e^	n-P (mg/g) ^f^
*E. carinatus sochureki*	2.57 (2.24–2.94)	2.5LD_50_	3.75 (3.43–4.10)	35.98 (31.36–41.16)	400	21.59	562.24
*E. carinatus multisquamatus*	1.57 (1.28–1.92)	2.5LD_50_	14.14 (10.82–18.46)	5.83 (4.75–7.13)	110	3.50	91.15
*D. russelii*	0.19 # (0.17–0.25)	5LD_50_	14.14 (10.82–18.46)	1.41 (1.26–1.86)	280	1.13	29.43

^a^ Median lethal dose: Dose of venom (µg/g) at which 50% of mice died. ^b^ Median effective dose: Dose of antivenom (µL) at which 50% of mice survived. ^c^ Median effective ratio: Ratio of venom (mg) to the volume of antivenom (ml) at which 50% of mice survived. ^d^ Potency: Number of LD_50_ completely neutralized per unit volume of antivenom (mL). ^e^ Potency: Amount of venom (mg) completely neutralized per unit volume of antivenom (mL). ^f^ Normalized potency: Amount of venom (mg) completely neutralized per unit amount of antivenom protein (g) for potency (P). # Value as reported by Faisal et al. [10].

## Data Availability

Not applicable.

## Supplementary Materials

The following supporting information can be downloaded at: https://www.mdpi.com/article/10.3390/toxins15040265/s1, Figure S1: (a) Replicate 1: PVAV concentration = 35.7 mg/mL; (b) Replicate 2: PVAV concentration = 36.3 mg/mL; (c) Replicate 3: PVAV concentration = 43.1 mg/mL; Table S1: Comparison of neutralizing efficacies of different antivenoms against lethality of Pakistani *D. russelii, E. carinatus sochureki*, and *E. carinatus multisquamatus*. Supplementary File S1: PVAV protein scores, mass spectral data (intensities, masses, and charges of ions) and amino acid sequences.
