# Supplementary material for: Proteomic Analysis, Immuno-Specificity and Neutralization Efficacy of Pakistani Viper Antivenom (PVAV), a Bivalent Anti-Viperid Antivenom Produced in Pakistan
Source: Toxins (Basel). 2023 Apr 3;15(4):265. doi: 10.3390/toxins15040265 (PMC10145215; doi:10.3390/toxins15040265)
Supplement: Supplementary file 1 [file toxins-15-00265-s001.zip › toxins-2304316 supplementary material/toxins-2304316 supplementary-merged Figure S1 & Table S1.pdf]

toxins

MDPI

Article

Proteomic Analysis, Immuno-Specificity and Neutralization Efficacy of Pakistani Viper Antivenom (PVAV), a Bivalent Anti-Viperid Antivenom Produced in Pakistan

Andy Shing Seng Lim, Kae Yi Tan, Naeem H. Quraishi, Saud Farooque, Zahoor Ahmed Khoso, Kavi Ratanabanangkoon and Choo Hock Tan

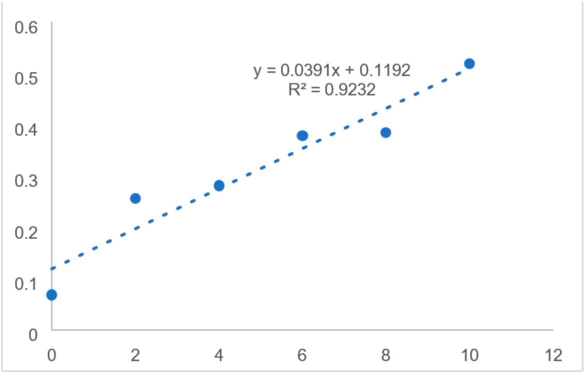

(a)

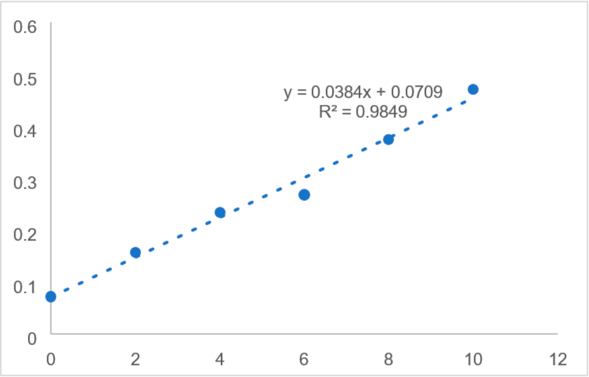

(b)

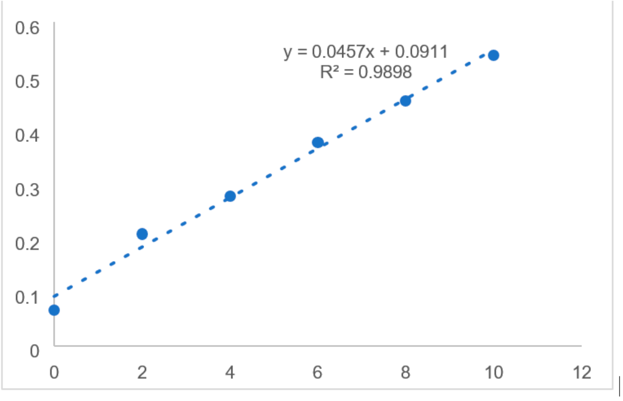

(c)

Figure S1. (**a**) Replicate 1: PVAV concentration = 35.7 mg/mL; (**b**) Replicate 2: PVAV concentration = 36.3 mg/mL; (**c**) Replicate 3: PVAV concentration = 43.1 mg/mL.

Table S1. Comparison of neutralizing efficacies of different antivenoms against lethality of Pakistani *D. russelii*, *E. carinatus sochureki*, and *E. carinatus multisquamatus*.

**Reference**	Current study		Faisal *et al.*, 2018 [10]		Pla *et al.*, 2019 [13]		Pla *et al.*, 2019 [13]		Pla *et al.*, 2019 [13]	
**Country of production**	Pakistan		Hyderabad, India		Hyderabad, India		San José, Costa Rica		Narayangaon, Maharashtra, India	
**Species against**	*E. carinatus sochureki*, *D. russelii* (Pakistan)		*Naja naja* (India), *Bungarus caeruleus, Daboia russelii* (India), *Echis carinatus carinatus*		*Naja naja* (India), *Bungarus caeruleus, Daboia russelii* (India), *Echis carinatus carinatus*		*Daboia russelii* (Sri Lanka), *Echis carinatus sinhaleyus, Hypnale hypnale, Naja naja* (Sri Lanka)		*Naja naja* (India), *Bungarus caeruleus, Daboia russelii* (India), *Echis carinatus carinatus*	
**Concentration**	38.4 mg/ml		84.9 mg/ml		55.5 mg/ml		58.0 mg/ml		35.7 mg/ml	
**Potency/normalized potency**	P (mg/ml)[a]	n-P (mg/g)[b]	P (mg/ml)[a]	n-P (mg/g)[b]	P (mg/ml)[a]	n-P (mg/g)[b]	P (mg/ml)[a]	n-P (mg/g)[b]	P (mg/ml)[a]	n-P (mg/g)[b]
D. russelii	1.13	29.43	0.23	2.70	1.24	22.32	1.53	25.62	1.78	28.34
E. carinatus sochureki	21.59	562.24	N.A.	N.A.	N.A.	N.A.	N.A.	N.A.	N.A.	N.A.
E. carinatus multisquamatus	3.50	91.15	N.A.	N.A.	N.A.	N.A.	N.A.	N.A.	N.A.	N.A.

Abbreviation: PVAV, Pakistani Viper Antivenom; VPAV, VINS Polyvalent Antivenom; ICP, Instituto Clodomiro Picado; N.A., Not applicable (not tested). [a] Potency: Amount of venom (mg) completely neutralized per unit volume of antivenom (ml). [b] Normalized potency: Amount of venom (mg) completely neutralized per unit amount of antivenom protein (g).
